# Reference values for cardiopulmonary exercise testing-derived parameters for cardiorespiratory fitness in Dutch community-dwelling 55- to 75-year-old adults

**DOI:** 10.1007/s00421-025-05978-w

**Published:** 2025-09-18

**Authors:** Dax Houtkamp, Annelies L. Pool-Goudzwaard, Tim Takken, Sabrina Chettouf, Albert Van de Wiel, Ivan Bautmans, Bart C. Bongers

**Affiliations:** 1 SOMT University of Physiotherapy, Amersfoort, The Netherlands; 2 Faculty of Behavioural and Movement Sciences, Amsterdam Movement Sciences, Vrije Universiteit Amsterdam, Amsterdam, The Netherlands; 3 Department of Medical Physiology, Child Development and Exercise Center, University Medical Center Utrecht, Utrecht, The Netherlands; 4 Faculty of Applied Sciences, Radiation Science and Technology, Clinical Medicine and Isotopes for Health, Delft University of Technology, Delft, The Netherlands; 5 Gerontology Department, Vrije Universiteit Brussel (VUB), Brussels, Belgium; 6 Frailty and Resilience in Ageing (FRIA) Research Unit, Vitality Research Group, Vrije Universiteit Brussel (VUB), Brussels, Belgium; 7 Geriatrics Department, Universitair Ziekenhuis Brussel (UZ Brussel), Brussels, Belgium; 8 Department of Nutrition and Movement Sciences, Institute of Nutrition and Translational Research in Metabolism (NUTRIM), Maastricht University, Maastricht, The Netherlands; 9 Department of Surgery, Institute of Nutrition and Translational Research in Metabolism (NUTRIM), Maastricht University, Maastricht, The Netherlands

**Keywords:** Cardiopulmonary exercise test, Reference values, Aging, Oxygen consumption, Anaerobic threshold, Physical fitness

## Abstract

**Purpose:**

Accurate interpretation of cardiorespiratory fitness (CRF) requires reference values that account for sex, age, and body composition. Existing reference values often lack these distinctions or exclude older adults. This study aimed to establish sex- and age-specific reference values for absolute and relative (body mass-corrected and lean body mass-corrected) CRF parameters derived from cardiopulmonary exercise testing (CPET) in Dutch community-dwelling 55- to 75-year-old adults.

**Methods:**

Cross-sectional data from 611 participants of the AMCOHF study were analyzed. CRF was assessed via cycle ergometer CPET evaluating oxygen uptake (V̇O_2peak_) and work rate (WR_peak_) at peak exercise, oxygen uptake at the ventilatory anaerobic threshold (V̇O_2VAT_), and oxygen uptake efficiency slope (OUES). Body mass and lean body mass were measured using dual-energy X-ray absorptiometry. Reference values stratified by sex and age were developed using generalized additive models. Prediction equations were generated using multiple linear regression. Correlations with V̇O_2peak_ assessed the usefulness of V̇O_2VAT_ and OUES as submaximal and effort-independent alternatives for CRF.

**Results:**

All CRF variables declined with age. V̇O_2peak_ (L/min) declined quasi-linearly (females: 1.3%/year; males: 2.5%/year). Significant sex differences were observed between all CRF variables (absolute and body mass-corrected values: *p* < 0.001; lean body mass-corrected values: *p* < 0.05). Significant correlations were found between V̇O_2peak_ and WR_peak_ (*ρ* = 0.90), V̇O_2VAT_ (*ρ* = 0.78), and OUES (*ρ* = 0.87).

**Conclusion:**

This study provides reference values for V̇O_2peak_, WR_peak_, V̇O_2VAT_, and OUES in Dutch older adults aged 55–75 years during cycle ergometer CPET, offering a unique dataset for assessing CRF and monitoring intervention effects.

**Supplementary Information:**

The online version contains supplementary material available at 10.1007/s00421-025-05978-w.

## Introduction

Low cardiorespiratory fitness (CRF) is a key predictor of adverse health outcomes in numerous chronic diseases, such as type 2 diabetes and cardiovascular disease, and all-cause mortality (Lang et al. 2024; Myers et al. 2024). CRF reflects the body's ability to transport oxygen from the lungs to muscle mitochondria to perform large muscle physical activity, thereby representing the integrated function of the cardiovascular, pulmonary, and musculoskeletal systems as a clinical vital sign (Ross et al. 2016). Moderate-to-high CRF levels are associated with lower chronic disease and mortality risks and the most significant health gains are observed when moving from the lowest fitness group to a higher level (McKinney et al. 2016; Sui et al. 2022). Identifying low CRF in older adults is especially important, as measuring a low CRF at an early stage can predict the risk of developing chronic diseases later in life (Myers et al. 2024). Moreover, a low CRF has been associated with reduced tolerance to medical treatment (e.g., perioperative risk, chemotherapy and/or radiation intolerance) (Levett et al. 2018). Therefore, if identified at an early stage, preventive strategies may be timely initiated to mitigate the risk of a low CRF, thereby preventing negative health outcomes.

Cardiopulmonary exercise testing (CPET) is the gold standard for measuring CRF, assessing oxygen uptake (V̇O_2_) at peak exercise (V̇O_2peak_) (Mezzani et al. 2009). Although V̇O_2peak_ is the primary indicator of CRF, many older adults fail to reach a true V̇O_2peak_ due to motivation and age-related limitations (Sanada et al. 2007; Wagner et al. 2020). Therefore, submaximal measures, such as the V̇O_2_ at the ventilatory anaerobic threshold (V̇O_2VAT_) that marks the onset of a significant contribution of anaerobic metabolism, and the effort-independent oxygen uptake efficiency slope (OUES), which assesses ventilatory efficiency, seem to be good alternative CRF indicators for older adults. Both V̇O_2VAT_ and OUES have demonstrated to be valid surrogates for V̇O_2peak_ in older adults and can therefore be used when V̇O_2peak_ is unattainable (Albouaini et al. 2007; Bongers et al. 2017).

To interpret an individual’s CRF correctly, adequate sex- and age-specific reference values for CRF, corrected for anthropometric characteristics (e.g., body height, body mass, lean body mass), are required (Ross 2003). Specifically, it has been shown that V̇O_2peak_ corrected for lean body mass is the most accurate expression of CRF when available (Imboden et al. 2020). Despite its importance, valid reference values for V̇O_2peak_, WR_peak_, V̇O_2VAT_, and OUES in older adults remain limited, often due to a sample without older adults (Buys et al. 2015; Van de Poppe et al. 2018; Van der Steeg and Takken 2021) or studies using an estimation for CRF, rather than measuring respiratory gas analysis directly, which has shown to provide errors in interpretation of CRF (Peterman et al. 2021). Finally, criteria for a maximal effort are often set too low (i.e., respiratory exchange ratio at peak exercise (RER_peak_) > 1.00), or not in line with recent suggestions to evaluate maximal effort based on age-dependent cut-off points (Wagner et al. 2020).

This study aimed to establish reference values for absolute V̇O_2peak_, work rate at peak exercise (WR_peak_), V̇O_2VAT_, and OUES in Dutch adults aged 55–75 years, as well as corrected for body mass and lean body mass. Reference values will be provided separately for females and males across the whole age range to examine the effects of sex and age on CRF. Males are expected to show higher values than females, and younger participants are expected to have higher values than older participants (van der Steeg and Takken 2021). Correlations between V̇O_2peak_ and other CRF measures (i.e., WR_peak_, V̇O_2VAT_, and OUES) will also be analyzed to assess their usefulness as alternatives to V̇O_2peak_. High correlations are expected between all CRF variables.

## Methods

### Experimental design

This cross-sectional study utilized data from the AMersfoort COhort Study on functional decline, Healthy aging, and Frailty (AMCOHF). Ethical approval was granted by the Medical Ethical Committee Zuyderland (Heerlen, The Netherlands) under reference number NL70141.096.19 (19 September 2019) and patient inclusion and data collection started in June 2022. The trial protocol has been published elsewhere (Houtkamp et al. 2025) and is registered on Open Science Framework (10.17605/OSF.IO/RMBQV).

### Participants

Participants were Dutch community-dwelling older adults aged 55–75 years from the city of Amersfoort, The Netherlands. Inclusion required informed consent, followed by a physician-led medical screening to rule out contraindications for maximal exercise testing. This screening included medical history and medication review, auscultation, baseline measures of blood pressure, and resting electrocardiography. Comorbidities were documented using the Rockwood Frailty Index as operationalized by Collerton et al. (2012).

Participants were excluded for the current study if they reported comorbidities to the physician that are likely to limit exercise capacity, including severe cardiovascular disease (e.g., ischemic heart disease, cerebrovascular disease, peripheral vascular disease, or heart failure). Participants who reported to suffer from chronic lung disease (e.g., chronic obstructive pulmonary disease or asthma) were excluded if the ratio between the forced expiratory volume in 1 s and forced vital capacity (Tiffeneau index) was < 0.70 (Mannino et al. 2007). Additionally, exclusion criteria for the AMCOHF cohort study involved active cancer, significant physical or cognitive impairment (i.e., mini mental state examination score < 23) according to previously described recommendations (Folstein et al. 1975), and having undergone surgery, cancer, chemotherapy, or radiotherapy in the last 6 months.

### Procedures

#### Cardiorespiratory fitness assessment

CRF was assessed using incremental CPET on a cycle ergometer (Lode Corival Rehab, Lode BV, Groningen, The Netherlands). Participants wore a facemask (Hans Rudolph, Kansas City, MO, USA) connected to an ergospirometry system (Metalyzer 3B, Cortex, Leipzig, Germany), calibrated for respiratory gas analysis and volume measurements. The Cortex Metalyzer 3B exhibited a measurement error of 2.85 ± 2.22% (Van Hooren et al. 2024). Forced vital capacity and forced expiratory volume in 1 s were measured before testing. Baseline cardiopulmonary values were recorded over a 3-min rest period, followed by a 3-min unloaded cycling warm-up. The work rate was then incrementally increased by 15–30 W/min according to a ramp protocol, tailored to each participant’s estimated fitness level to achieve maximal effort within 8–12 min (Glaab and Taube 2022). Participants maintained a pedaling frequency between 60 and 80 rotations/min until they reached exhaustion despite verbal encouragement or until they reached criteria for termination based on the American Thoracic Society/American College of Chest Physicians guidelines (2003). After termination of the incremental protocol, participants proceeded in a 1-min cooldown period consisting of a constant load of 50 W. Breath-by-breath V̇O_2_, carbon dioxide production (V̇CO_2_), and respiratory exchange ratio (RER) were calculated and averaged over 10-s intervals. Heart rate (HR) was continuously monitored by 12-lead electrocardiography. Our study protocol aimed to collect CRF reference data using the quality criteria for CPET standards as defined previously (Takken et al. 2019).

#### Body composition assessment

Body height and body mass were measured to the nearest 0.1 cm and 0.1 kg, respectively, and body mass index (BMI) was calculated subsequently. Lean body mass was evaluated using a fan beam dual-energy X-ray absorptiometry (DXA) device (GE Medical Systems Lunar Prodigy, Madison, Wisconsin, USA). This enables the determination of lean body mass and whole-body fat percentage and is considered highly accurate (Shepherd et al. 2017). Waist circumference was measured to compare body composition with previous studies.

### Data analysis

For descriptive analyses, participants were divided in 5-year age groups except for the last age group (i.e., 70–75 years) to ensure enough samples. A maximal cardiorespiratory effort during CPET was defined as reaching the age-dependent cut-off points for RER_peak_ (i.e., ≥ 1.10 for age group 55–59 or ≥ 1.06 for age group 60–75) or peak heart rate (≥ 92% of 208 – 0.7 × age for age group 55–59 or ≥ 89% for age group 60–75) (Tanaka et al. 2001; Wagner et al. 2020). The work rate at peak exercise (WR_peak_) was the highest attained value. Data from other outcome variables were averaged over 30 s of exercise. Non-maximal efforts were excluded from the analysis of V̇O_2peak_ and WR_peak_ but were used for analysis of submaximal or effort-independent CRF parameters (i.e., respectively V̇O_2VAT_ and OUES). The V̇O_2VAT_ was determined primarily using the modified V-slope method (i.e., the point at which the linear relationship between the V̇CO_2_ and V̇O_2_ changed). This point was verified using the ventilatory equivalents method (i.e., the point at which the ventilatory equivalent for oxygen and the partial end-tidal oxygen tension reached its lowest point after which it began to increase in a consistent manner, although the ventilatory equivalent for carbon dioxide and partial end-tidal carbon dioxide tension remained constant). Detailed description of the methods can be found in previously described guidelines (Franssen et al. 2022). Cases with ambiguous V̇O_2VAT_ were resolved by consensus with two experts (BB and DH). The oxygen uptake efficiency slope (OUES) was calculated using previously described formula (i.e., a × log_10_ V̇E + b, where “a” represents the OUES and “b” represents the intercept) (Baba et al. 1996). Data from 1 min after the start of the test up (i.e., to prevent the noisy V̇O_2_ at the start from influencing the OUES) to V̇O_2peak_. When a plateau in V̇O_2_ was detected, data up to the onset of the V̇O_2_ plateau were used to determine the OUES, in accordance with prior recommendations (Niemeijer et al. 2014).

Several other cardiorespiratory parameters were determined (i.e., RER, heart rate, oxygen pulse, minute ventilation, tidal volume, and breathing frequency at peak exercise, and the slope between the minute ventilation and V̇CO_2_). The slope between the minute ventilation and V̇CO_2_ (V̇E/V̇CO_2_-slope) was calculated up to the respiratory compensation point (RCP) which was determined using the interpretative guidelines as described before (Franssen et al. 2022). Cases with ambiguous RCP were resolved by consensus of the same two experts (BB and DH). When the RCP was not visible, the V̇E/V̇CO_2_ slope was calculated using data up to peak exercise.

CPETs with insufficient data quality or implausible response patterns were independently evaluated by two experts (BB and DH) and excluded from the analysis if consensus was reached that the test was inaccurate.

### Statistical analysis

Statistical analyses were conducted using R version 4.4.2. (R Foundation for Statistical Computing, Vienna, Austria). Data are presented as mean (SD) and statistical significance was set at *p* < 0.05. Independent t tests assessed sex differences, while age effects were evaluated using one-way ANOVA with Bonferroni post hoc adjustments when needed. To assess the relationships between CPET-derived CRF variables (i.e., V̇O_2peak_, WR_peak_, V̇O_2VAT_, and OUES), Pearson’s correlation coefficients were calculated. In cases where the assumptions of normality were not met, Spearman’s rank coefficients were used. Correlation strength was interpreted as follows: *r* < 0.30 = weak, 0.30–0.59 = moderate, and ≥ 0.60 = strong (Cohen 2013). Reference centiles (P3, P10, P25, P50, P75, P90, P97) were derived using generalized additive models (GAM) which has previously shown to be the best fitting with highest predictive accuracy compared to linear or polynomial model (Mylius et al. 2019). Model performance of the GAM was assessed using adjusted R^2^. All body mass- and lean body mass-corrected CRF parameters were obtained by dividing absolute values by body mass and lean body mass. This was done to facilitate comparisons across differences in body size and body composition, respectively. Descriptive statistics were calculated by 5-year age groups and sex.

## Results

**Fig. 1:**
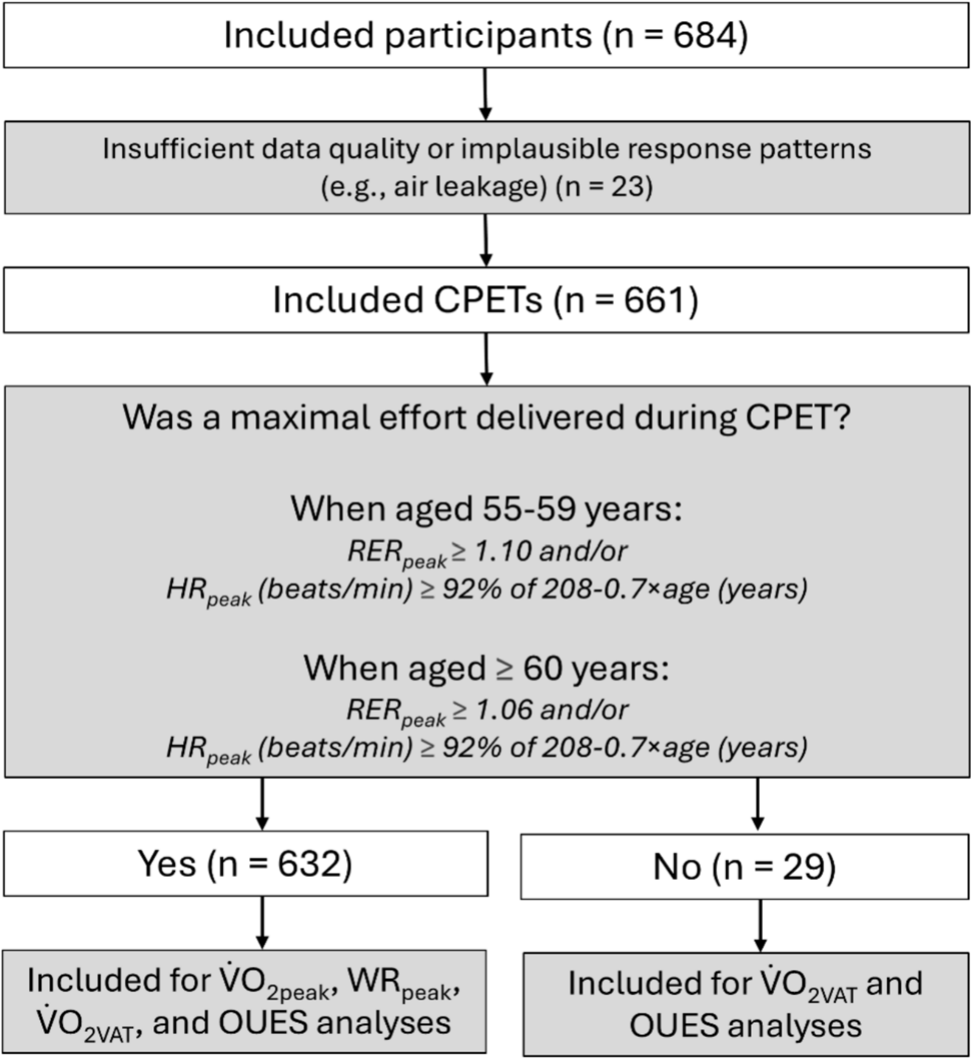
Flowchart depicting the study’s data analysis process. *CPET* cardiopulmonary exercise testing, *HR*_*peak*_ heart rate at peak exercise, *OUES* oxygen uptake efficiency slope, *RER*_*peak*_ respiratory exchange ratio at peak exercise, *V̇O*_*2VAT*_ oxygen uptake at the ventilatory anaerobic threshold, *V̇O*_*2peak*_ oxygen uptake at peak exercise, *WR*_*peak*_, work rate at peak exercise

A total of 661 subjects were included in the final analysis (336 females and 325 males). Participants were equally distributed across 5-year intervals from 55 to 75 years, with at least 50 participants representing each age group. A flow diagram of the study is shown in Fig. 1. Participant characteristics, stratified by sex and age group and including experimental CPET results (i.e., V̇O_2peak_, WR_peak_, V̇O_2VAT_, and OUES), are presented in Table 1.

**Table 1:** Participant characteristics and experimental CPET results of the study population

Variable	Total (*n* = 661)	Females (*n* = 336)				Males (*n* = 325)			
		55–59 (*n* = 73)	60–64 (*n* = 98)	65–69 (*n* = 107)	70–75 (*n* = 58)	55–59 (*n* = 50)	60–64 (*n* = 77)	65–69 (*n* = 103)	70–75 (*n* = 95)
Body height (cm)	174 (9)	169 (5)	169 (5)	167 (6)	165 (6)	182 (7)	183 (7)	180 (7)	180 (6)
Body mass (kg)	78 (14)	74 (12)	72 (13)	69 (11)	67 (8)	89 (13)	86 (11)	85 (13)	83 (10)
BMI (kg/m^2^)	25.5 (3.6)	25.9 (4.1)	25.3 (4.3)	24.7 (4.0)	24.3 (2.7)	26.7 (3.6)	25.9 (3.5)	25.9 (3.4)	25.8 (2.7)
Lean body mass (kg)	51 (10)	44 (5)	43 (4)	42 (4)	41 (3)	62 (7)	61 (6)	59 (6)	59 (7)
% Body fat	30.3 (8.8)	36.4 (8.7)	35.9 (7.5)	34.4 (8.3)	35.1 (6.5)	24.6 (7.1)	25.0 (5.6)	25.3 (6.4)	25.3 (6.2)
Waist circumference (cm)	94 (10)	91 (11)	92 (11)	92 (11)	90 (9)	98 (12)	97 (8)	97 (9)	97 (7)
DBP (mmHg)	80 (7)	79 (7)	79 (7)	80 (9)	80 (6)	80 (5)	80 (7)	80 (8)	81 (7)
SBP (mmHg)	131 (17)	126 (14)	126 (16)	133 (19)	132 (15)	128 (13)	128 (14)	133 (16)	140 (18)
# Higher education (%)	64	61	49	60	48	84	76	75	65
# Smoking (%)	4.6	2.9	2.1	2.0	1.7	4.3	5.6	6.2	4.4
WR_peak_ (W)^a^	200 (51)	184 (29)	171 (27)	162 (23)	146 (23)	280 (48)	249 (32)	229 (35)	208 (36)
V̇O_2peak_ (L/min)^a^	2.29 (0.6)	2.10 (0.38)	1.96 (0.36)	1.86 (0.29)	1.69 (0.27)	3.14 (0.58)	2.89 (0.43)	2.62 (0.43)	2.38 (0.47)
V̇O_2VAT_ (L/min)	1.33 (0.34)	1.22 (0.31)	1.20 (0.24)	1.16 (0.22)	1.10 (0.2)	1.58 (0.41)	1.55 (0.29)	1.50 (0.33)	1.40 (0.35)
V̇O_2VAT_/V̇O_2peak_ (%)^a^	59 (10)	59 (8)	61 (7)	64 (9)	66 (9)	51 (9)	54 (7)	58 (10)	59 (9)
OUES	2.63 (0.69)	2.39 (0.57)	2.26 (0.46)	2.19 (0.38)	2.02 (0.39)	3.35 (0.75)	3.27 (0.54)	2.96 (0.53)	2.80 (0.61)
V̇E/V̇CO_2_-slope	29.1 (4.5)	27.5 (4.0)	28.3 (4.7)	28.8 (4.1)	30.2 (4.7)	29.4 (4.0)	28.1 (4.3)	29.4 (4.6)	30.9 (4.7)
RER_peak_	1.10 (0.08)	1.11 (0.07)	1.10 (0.07)	1.09 (0.08)	1.07 (0.07)	1.13 (0.08)	1.12 (0.07)	1.12 (0.07)	1.09 (0.08)
HR_peak_ (beats/min)	158 (14)	162 (14)	160 (13)	157 (13)	151 (13)	163 (15)	161 (15)	158 (14)	153 (16)
O_2_-pulse_peak_ (mL/beat)	16.0 (3.9)	13.6 (3.4)	12.7 (2.4)	12.1 (2.3)	11.5 (2.0)	19.3 (3.8)	18.4 (2.8)	17.0 (2.7)	16.2 (3.3)
V̇E_peak_ (L/min)	88 (28)	75 (16)	72 (19)	68 (16)	64 (15)	124 (27)	112 (26)	103 (22)	94 (23)
VT_peak_ (L)	2.4 (0.7)	2.1 (0.4)	2.0 (0.4)	1.9 (0.4)	1.8 (0.4)	3.3 (0.5)	3.0 (0.5)	2.9 (0.5)	2.7 (0.5)
BF_peak_ (breaths/min)	36 (7)	36 (7)	36 (7)	36 (7)	35 (7)	38 (8)	38 (8)	36 (6)	36 (8)

Results are quantified as mean (SD)

*BF*_*peak*_ breathing frequency at peak exercise; *BMI* body mass index; *DBP* diastolic blood pressure; *HR*_*peak*_ heart rate at peak exercise; *RER*_*peak*_ respiratory exchange ratio at peak exercise; *O*_*2*_*-pulse*_*peak*_ oxygen pulse at peak exercise; *SBP* systolic blood pressure; *VE/VCO*_*2*_* slope* slope between the minute ventilation and carbon dioxide production up to the respiratory compensation point; *V̇E*_*peak*_ minute ventilation at peak exercise; *V̇O*_*2peak*_ oxygen uptake at peak exercise; *V̇O*_*2VAT*_ oxygen uptake at the ventilatory anaerobic threshold; *OUES* oxygen uptake efficiency slope; *VT*_*peak*_ tidal volume at peak exercise; *WR*_*peak*_ work rate at peak exercise

^a^*n* = 632 for V̇O_2peak_, WR_peak_, and V̇O_2VAT_/V̇O_2peak_, as 29 participants did not perform a maximal cardiorespiratory effort

### Cardiopulmonary exercise testing-derived parameters of cardiorespiratory fitness

**Fig. 2:**
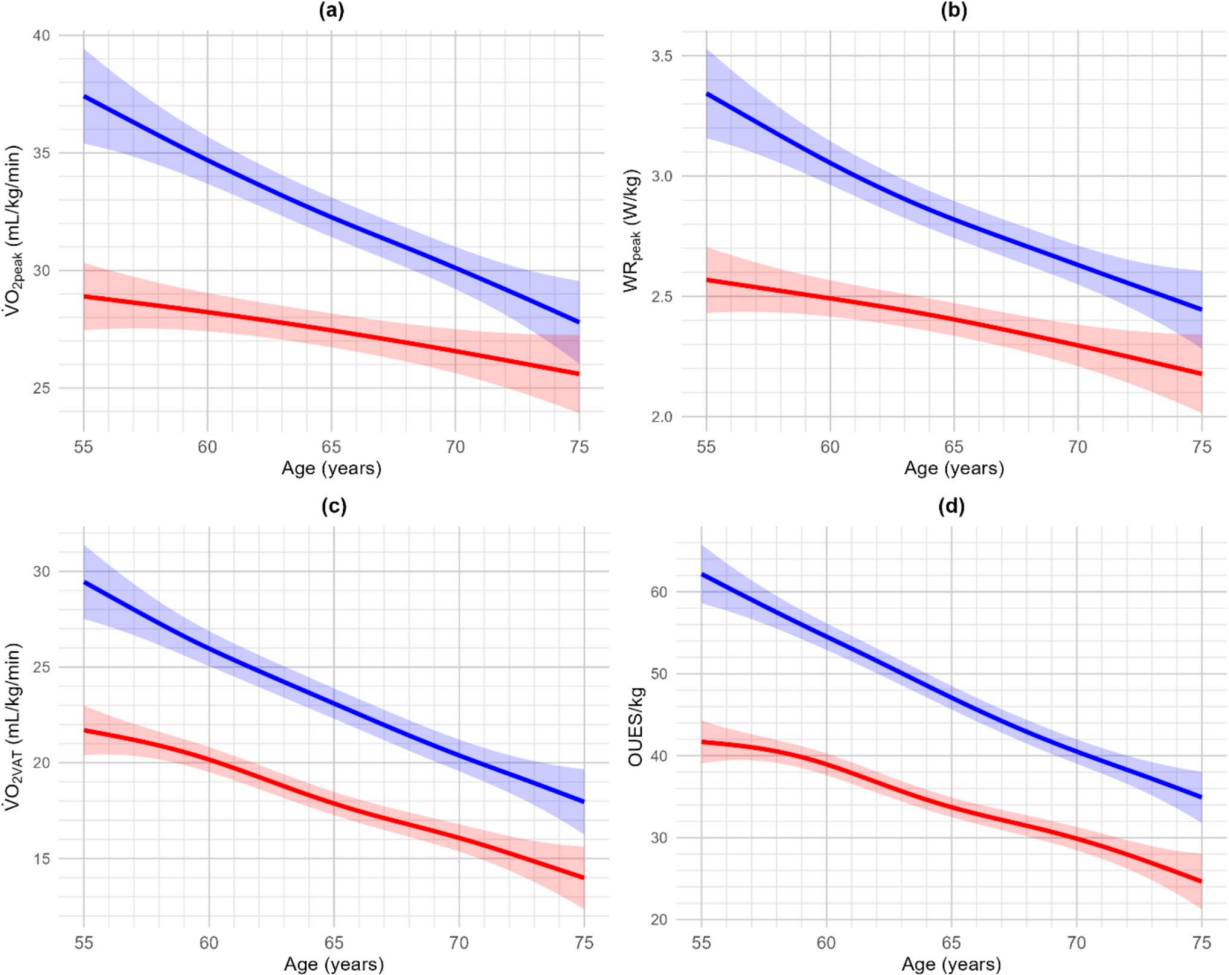
Age-dependent transformation of **a** oxygen uptake at peak exercise, **b** work rate at peak exercise, **c** oxygen uptake at the ventilatory threshold, and **d** oxygen uptake efficiency slope corrected for body mass in females (red) and males (blue) using generalized additive models. Shading represents 95% confidence interval. *OUES* oxygen uptake efficiency slope, *V̇O*_*2VAT*_ oxygen uptake at the ventilatory anaerobic threshold, *V̇O*_*2peak*_ oxygen uptake at peak exercise, *WR*_*peak*_ work rate at peak exercise

Reference values for CPET-derived CRF parameters, corrected for body mass and estimated using GAM by sex and 5-year age groups, are presented in Table 2 for maximal CRF variables (i.e., V̇O_2peak_ and WR_peak_) and in Table 3 for submaximal (i.e., V̇O_2VAT_) and effort-independent (i.e., OUES) variables. Reference values with age as continuous variable are shown in Fig. 2. Similar analyses indexed for absolute reference values and values corrected for lean body mass are available in Online Resource 1.

**Table 2:** Additive models of maximal CRF parameters (V̇O_2peak_ and WR_peak_) corrected for body mass for females (*n* = 323)^a^ and males (*n* = 309)^a^ in the different age groups

Age	Body height	Body mass	Percentiles						
	(cm)	(kg)	3	10	25	50	75	90	97
V̇O_2peak_ (mL/kg/min)									
*Females*									
55–59	169	73.3	20.2	23.1	26.0	29.3	32.5	35.4	38.3
60–64	168	69.9	19.3	22.2	25.1	28.4	32.6	34.5	37.4
65–69	166	67.6	18.3	21.1	24.1	27.3	30.5	33.5	36.3
70–75	165	66.6	16.9	19.7	22.7	25.9	29.1	32.1	34.9
*Males*									
55–59	183	87.4	27.2	30.0	33.0	36.2	39.4	42.4	45.2
60–64	182	85.4	24.8	27.7	30.6	33.8	37.1	40.0	42.9
65–69	180	83.9	22.3	25.1	28.1	31.3	34.5	37.5	40.3
70–75	180	83.2	19.7	22.6	25.5	28.8	32.0	34.9	38.8
WR_peak_ (W/kg)									
*Females*									
55–59	169	73.3	1.87	2.10	2.33	2.59	2.84	3.07	3.30
60–64	168	69.9	1.79	2.01	2.25	2.50	2.76	2.99	3.22
65–69	166	67.6	1.67	1.90	2.13	2.38	2.64	2.87	3.10
70–75	165	66.6	1.50	1.73	1.96	2.22	2.47	2.70	3.93
*Males*									
55–59	183	87.4	2.53	2.76	3.99	3.25	3.50	3.73	3.96
60–64	182	85.4	2.25	2.48	2.71	2.96	3.22	3.45	3.68
65–69	180	83.9	2.01	2.24	2.47	2.73	2.98	3.21	3.44
70–75	180	83.2	1.82	2.04	2.28	2.53	2.79	3.02	3.25

*CRF* cardiorespiratory fitness; *V̇O*_*2peak*_ oxygen uptake at peak exercise; *WR*_*peak*_ work rate at peak exercise

^a^For V̇O_2peak_ and WR_peak_ *n* = 632, because 29 participants did not perform a maximal cardiorespiratory effort

**Table 3:** Additive models of submaximal CRF parameters (V̇O_2VAT_ and OUES) corrected for body mass for females (*n* = 336) and males (*n* = 325) in the different age groups

Age	Body height	Body mass	Percentiles						
	(cm)	(kg)	3	10	25	50	75	90	97
V̇O_2VAT_ (mL/kg/min)									
*Females*									
55–59	169	73.3	12.6	15.2	17.9	19.9	23.7	26.4	29.0
60–64	168	69.9	10.8	13.4	16.0	18.0	21.9	24.5	27.2
65–69	166	67.6	8.7	11.3	13.9	16.6	19.8	22.4	25.0
70–75	165	66.6	6.8	9.4	12.1	15.0	17.9	20.1	23.2
*Males*									
55–59	183	87.4	18.9	21.5	24.1	27.0	30.0	32.6	35.2
60–64	182	85.4	15.2	17.8	20.5	23.2	26.3	29.0	31.6
65–69	180	83.9	12.8	15.4	18.0	20.8	23.9	26.5	29.1
70–75	180	83.2	9.8	12.4	15.0	17.9	20.9	23.5	26.9
OUES/kg									
*Females*									
55–59	169	73.3	25.4	30.0	34.7	39.9	45.1	49.8	54.4
60–64	168	69.9	21.6	26.3	30.9	36.18	41.3	45.0	50.6
65–69	166	67.6	17.0	21.6	26.3	31.5	36.7	41.3	46.0
70–75	165	66.6	12.7	17.3	22.0	27.2	32.4	37.0	41.7
*Males*									
55–59	183	87.4	43.5	48.1	52.8	56.3	63.2	67.9	72.5
60–64	182	85.4	36.2	40.9	45.5	50.7	55.9	60.6	72.9
65–69	180	83.9	29.3	33.9	38.6	43.8	49.0	53.7	58.3
70–75	180	83.2	23.5	28.1	32.8	38.0	43.2	47.9	52.5

*CRF* cardiorespiratory fitness; *OUES* oxygen uptake efficiency slope; *V̇O*_*2VAT*_ oxygen uptake at the ventilatory anaerobic threshold

### Effects of sex and age on cardiorespiratory fitness

All absolute and body mass-corrected CPET-derived CRF variables were significantly higher in males compared to females across all age groups (*p* < 0.001), but not when corrected for lean body mass except for WR_peak_ (*p* = 0.027). Additionally, increasing age was associated with a decline in all CRF variables (*p* = 0.048 for V̇O_2VAT_ corrected for lean body mass; all other *p* values: *p* < 0.001).

### Correlations between variables of cardiorespiratory fitness

Spearman correlation coefficients were calculated between all absolute CRF variables. The analyses revealed significant positive correlations between V̇O_2peak_ and, respectively, WR_peak_ (*ρ* = 0.90, *p* < 0.001), V̇O_2VAT_ (*ρ* = 0.78, *p* < 0.001), and OUES (*ρ* = 0.87, *p* < 0.001), indicating strong associations between these CPET-derived variables in older adults.

### Reference values

Prediction equations to calculate absolute and body mass-corrected CRF parameters were developed using multiple linear regression models and can be found in Table 4. Covariates include sex (0 = female, 1 = male) and three continuous variables (i.e., age, body height, and body mass).

**Table 4:** Prediction equations for CRF parameters (V̇O_2peak_, WR_peak_, V̇O_2VAT_, and OUES), developed using multiple linear regression analysis to express CPET performance as a percentage of predicted, corrected for sex (0 = female; 1 = male), age (years), body height (cm), and body mass (kg)

CRF variable	Prediction equation	Adjusted R^2^
V̇O_2peak_ (L/min)	1.538 + (0.292 × *sex*) – (0.022 × *age*) + (0.009 × *body height*) + (0.003 × *body mass*) – (0.023 × *sex* × *age*) + (0.009 × *sex* × *body height*) + (0.002 × *sex* × *body mass*)	0.608
V̇O_2peak_ (mL/kg/min)	48.721 + (2.539 × *sex*) – (0.322 × a*ge*) + (0.126 × *body height*) – (0.308 × *body mass*- (0.204 × *sex* × *age*) + (0.077 × *sex* × *body height*) + (0.056 × *sex* × *body mass*)	0.428
WR_peak_ (W)	89.371 + (72.772 × *sex*) – (2.281 × *age*) + (1.428 × *body height*) – (0.236 × *body mass*) – (1.994 × *sex* × *age*) + (0.611 × *sex* × *body height*) + (0.059 × *sex* × *body mass*) 105.279 – 24.247 × *Sex* – 0.498 × *Age* – 0.123 × *Height* – 0.111 × *Body mass* – 0.186 × *Sex* × *Age* + 0.23 × *Sex* × *Height* – 0.012 × *Sex* × *Body mass*	0.666
WR_peak_ (W/kg)	3.747 + (0.908 × s*ex*) – (0.034 × *age*) + (0.019 × *body height*) – (0.034 × *body mass*) – (0.016 × s*ex* × *age*) + (0.002 × *sex* × *body height*) + (0.005 × *sex* × *body mass*)	0.551
V̇O_2VAT_ (L/min)	0.668 – (1.44 × *sex*) – (0.006 × *age*) + (0.004 × *body height*) + (0.002 × *body mass*) – (0.003 × *sex* × *age*) + (0.011 × *sex* × *body height*)	0.303
V̇O_2VAT_ (mL/kg/min)	27.829 – (16.141 × *sex*) – (0.363 × *age*) + (0.066 × *body height*) + (0.038 × *body mass*) – (0.124 × *sex* × *age*) + (0.16 × *sex* × *body height*) – (0.012 × *sex* × *body mass*)	0.396
OUES	0.91 – (0.726 × *sex*) – (0.017 × *age*) + (0.013 × *body height*) + (0.004 × *body mass*) – (0.015 × *sex* × *age*) + (0.01 × *sex* × *body height*) + (0.005 × *sex* × *body mass*)	0.485
OUES/kg	47.724 + (4.211 × *sex*) – (0.789 × *age*) + (0.197 × *body height*) + (0.066 × *body mass*) – (0.43 × *sex* × *age*) + (0.145 × *sex* × *body height*) + (0.085 × *sex* × *body mass*)	0.566

*CRF* cardiorespiratory fitness; *OUES* oxygen uptake efficiency slope; *V̇O*_*2peak*_ oxygen uptake at peak exercise; *V̇O*_*2VAT*_ oxygen uptake at the ventilatory anaerobic threshold; *WR*_*peak*_ work rate at peak exercise

## Discussion

This study provides reference values for maximal and submaximal CPET-derived parameters of CRF in Dutch community-dwelling older adults aged 55–75 years. In addition, results also show robust relationships between V̇O_2peak_ and surrogate measures of CRF (i.e., WR_peak_, V̇O_2VAT_, and OUES), highlighting their potential to evaluate CRF in older adults when respiratory gas analysis is unavailable (i.e., WR_peak_) or when a participant is not able to deliver a maximal effort (i.e., V̇O_2VAT_ and OUES). Furthermore, results showed that sex differences between all CRF variables could mainly be attributed to differences in lean body mass, as indicated by far greater differences in absolute values or values corrected for body mass compared to CRF parameters corrected for lean body mass (Online Resource 1). Therefore, it is recommended to correct CPET-derived CRF outcomes for lean body mass if resources allow.

To the authors’ knowledge, this is the first study to provide sex- and age-specific reference values for maximal (i.e., V̇O_2peak_ and WR_peak_) and submaximal (i.e., V̇O_2VAT_ and OUES) CPET-derived parameters of CRF in absolute terms, as well as corrected for body mass and lean body mass in older adults. Currently utilized Dutch reference values do not include participants up to an age of 75 years and are not based on such a large and well-characterized cohort, nor have they been developed using GAM, which has shown to outperform linear or polynomial models (Mylius et al. 2019). Additionally, including submaximal reference data are important for assessing CRF in older adults who are unable to perform a cardiorespiratory maximal effort during CPET.

V̇O_2peak_ declined on average by 1.3% (0.02 L/min) per year for females and 2.5% (0.07 L/min) per year for males. This decline can partly be attributed to changes in body composition, as the decline in V̇O_2peak_ was lower when corrected for body mass (females: 0.8% or 0.2 mL/kg/min per year; males: 1.5% or 0.5 mL/kg/min per year) or lean body mass (females: 0.9% or 0.4 mL/kg lean body mass/min per year; males: 1.5% or 0.7 mL/kg lean body mass/min per year). This illustrates that the decline in V̇O_2peak_ in older adults is comparable to the decline of approximately one percent per year over the course of a lifetime, as has been typically described (Kenney et al. 2022). Additionally, the decline in V̇O_2peak_ per year observed within the youngest age group was comparable with the decline observed in the oldest age group for females (55–60 years: 0.02 L/min or 1.1%; 70–75 years: 0.02 L/min or 1.2%) and in absolute terms for males (55–60 years: 0.08 L/min or 2.5%; 70–75 years: 0.09 L/min or 4.2%), although the relative decline (in percentage) was slightly higher in the oldest age group, highlighting a quasi-linear trend of decline.

### Comparison with other maximal reference data

Compared with previous Dutch references values, V̇O_2peak_ from the AMCOHF cohort was slightly lower (*p* < 0.05) than the previously reported Lowlands Fitness Registry data (males: − 1.4 mL/kg/min; females: − 1.6 mL/kg/min) for individuals around 55 years of age (van der Steeg and Takken 2021). These small differences support the potential integration of our data into the Lowlands Fitness Registry to extend the age range up to 75 years. Among adults aged 55–64 years, V̇O_2peak_ in the current study was slightly higher than in the German SHIP study (males: + 1.3 mL/kg/min; females: + 3.1 mL/kg/min; Koch et al. 2009), but slightly lower than in the Swiss COmPLETE study (males: − 1.7 mL/kg/min; females: − 0.4 mL/kg/min; Wagner et al. 2021). Both SHIP and COmPLETE cohorts consisted of healthy older adults, allowing a fair comparison with the AMCOHF cohort. Compared to reference values from the United States (Kaminsky et al. 2022), the current study reported substantially higher V̇O_2peak_ (males: + 10.9 mL/kg/min; females: + 11.7 mL/kg/min) and WR_peak_ (males: + 96 W; females: + 82 W) values in healthy adults around 55 years of age (*p* < 0.001 for all values). These differences may be explained by variations in CPET protocols, by a low RER_peak_ criterion to indicate a maximal effort (≥ 1.0), potentially resulting in submaximal V̇O_2peak_ values, as well as by the inclusion of participants with conditions, such as obesity, diabetes, and cardiovascular risk factors that could limit their tolerance to exercise.

In accordance to these findings, GAM-predicted WR_peak_ in this study was also significantly higher (*p* < 0.001) compared to previously described Dutch reference data by Van de Poppe et al. (2018), for age groups 55 (females: 187 vs 165 W; males: 293 vs 251 W) and 60 (females: 177 vs 148 W; males: 263 vs 221 W). However, data collection in that study was also not uniform across all measurements, which might explain part of these differences. The presented GAM-predicted OUES values in this study were slightly higher for females ($\Delta$OUES: 243, *p* < 0.05), but not for males ($\Delta$OUES: 158, *p* = 0.14) aged 55 and 60 years compared to previous reference values from Belgium (Buys et al. 2015). Although it has been well documented that V̇O_2peak_ and V̇O_2VAT_ decline with age, reference values in especially the highest age categories and in females are currently still lacking (van der Steeg and Takken 2021).

Our findings align with the previous literature demonstrating that the age-related CRF decline is partly mitigated when corrected for lean body mass (Kim et al. 2016). Furthermore, the recommendation of correcting V̇O_2peak_ for lean body mass when possible is in accordance to previous suggestions (Imboden et al. 2020; Köhler et al. 2018). Nevertheless, the decline in cardiovascular capacity has been described as one of the prominent factors to contribute to a decline in CRF (Kenney et al. 2022). In contrast to the correction for body mass, sex differences between all four CRF variables seem to disappear almost completely when looking at the CRF variables corrected for lean body mass. This in accordance with the previous literature demonstrating a higher fat percentage per given amount of body mass in females compared to males (Schorr et al. 2018).

### Strength and limitations

Our study protocol met 11 out of 14 quality criteria for CPET standards (Takken et al. 2019). Another strength is that this study used recently developed cut-off points for RER_peak_ to determine whether a maximal effort was reached (Wagner et al. 2020). These cut-off points were age-dependent, compared to a large number of studies using a RER_peak_ cut-off of ≥ 1.00, potentially leading to inclusion of submaximal cardiorespiratory efforts. Furthermore, these data were collected at one laboratory setting preventing heterogeneity between multiple testing facilities done in the previous studies. However, this study also has some limitations. Physical activity was not measured in this cohort, which limits direct comparison with other studies. Although people were randomly invited to participate in this study, a potential selection bias of healthy physically active people could have led to an overestimation of the CRF level of the participants. This may be substantiated by the relatively few smokers in our sample compared to the average Dutch population (van Aerde et al. 2024).

### Practical implications

Reference values for CRF are of pivotal importance, because they provide a benchmark to assess an individual’s health status, detect functional decline at an early stage, and guide personalized interventions for healthy aging. With the aging Dutch population, establishing adequate reference values for older adults is essential for clinical decision-making, for example in geriatric, cardiac, and pulmonary settings. Additionally, in sports medicine, reference data for apparently healthy older individuals are necessary, as the growing participation of master athletes in endurance events often requires medical evaluations, such as CPET, as part of the entry requirement to ensure safe participation. Applications include for example preoperative evaluations, helping to identify individuals at risk of postoperative complications, enabling targeted prehabilitation interventions to optimize surgical outcomes (Levett and Grocott 2025). Additionally, CPET-based assessments allow for early detection of a declining intrinsic capacity as proposed by the WHO. Without appropriate norm values, distinguishing between physiological aging and pathological declines in CRF remains challenging, potentially leading to inadequate risk assessment and suboptimal clinical decision-making.

The prediction equations for CRF presented in Table 4 are not intended as a substitute for direct objective measurement of CRF, but rather as a tool to express an individual’s CPET performance relative to expected values after accounting for sex, age, body height, and body mass. Future studies are warranted to validate these prediction equations in independent datasets.

The inclusion of corrections for both body mass and lean body mass enhances the applicability of this dataset by reducing misclassification due to age-related changes in body composition. Additionally, establishing reference values for submaximal parameters also enhances the applicability in older populations by providing benchmarks for a good, average, or poor CRF based on sex and age. Future studies presenting reference values should provide these based on longitudinal data. By monitoring the trajectory of an individual’s CRF, potential risk factors for a declining CRF may be discovered. In addition, physical activity data should be considered to make sure that no selection bias occurs. Finally, larger databases would allow for computation of body height- and body mass-specific reference values within sex- and age-specific groups.

## Conclusion

This study provides reference values for CRF in Dutch older adults aged between 55 and 75 years during cycle ergometer CPET. All CRF variables declined with age, with an average V̇O_2peak_ decline of 1.3% per year for females and 2.5% for males. Sex differences were present between all absolute and body mass-corrected CRF variables, but not when corrected for lean body mass. Furthermore, WR_peak_, V̇O_2VAT_, and OUES can be used as practical alternative measures for V̇O_2peak_. Together, these reference values can be used to estimate an individual’s CRF for decision-making in different clinical settings.

## Supplementary Information

Below is the link to the electronic supplementary material.

Supplementary file1 (DOCX 471 KB)

## Data Availability

All data supporting the findings of this study are available at the Open Science Framework repository (10.17605/OSF.IO/RMBQV). The study was conducted at a private institution without the use of public funding. Users of the dataset are kindly requested to inform the corresponding author if the data are used for additional analyses.

## Abbreviations

AMCOHF: AMersfoort COhort study on functional decline, Healthy aging, and Frailty
BF_peak_: Breathing frequency at peak exercise
BMI: Body mass index
CPET: Cardiopulmonary exercise testing
CRF: Cardiorespiratory fitness
DBP: Diastolic blood pressure
DXA: Dual-energy X-ray absorptiometry
GAM: Generalized additive models
HR: Heart rate
HR_peak_: Heart rate at peak exercise
O_2_-pulse_peak_: Oxygen pulse at peak exercise
OUES: Oxygen uptake efficiency slope
RCP: Respiratory compensation point
RER: Respiratory exchange ratio
RER_peak_: Respiratory exchange ratio at peak exercise
SBP: Systolic blood pressure
V̇CO_2_: Carbon dioxide production
VE/VCO_2_ slope: Slope between the minute ventilation and carbon dioxide production up to the respiratory compensation point
V̇E_peak_: Minute ventilation at peak exercise
V̇O_2_: Oxygen uptake
V̇O_2peak_: Oxygen uptake at peak exercise
V̇O_2VAT_: Oxygen uptake at the ventilatory anaerobic threshold
VT_peak_: Tidal volume at peak exercise
WR_peak_: Work rate at peak exercise
