# Supplementary material for: Reference values for cardiopulmonary exercise testing-derived parameters for cardiorespiratory fitness in Dutch community-dwelling 55- to 75-year-old adults
Source: Eur J Appl Physiol. 2025 Sep 18;126(2):1067–77. doi: 10.1007/s00421-025-05978-w (PMC12948906; doi:10.1007/s00421-025-05978-w)
Supplement: Supplementary file 1 — Supplementary file1 (DOCX 471 KB) [file 421_2025_5978_MOESM1_ESM.docx]

**Online Resource 1**

**Table 1 Additive models of maximal CRF parameters (**V̇**O_2peak_ and WR_peak_) for females (n = 323)^a^ and males (n = 309)^a^ in the different age groups.**

| Age | Body height | | Body mass | Percentiles | | | | | | | |
| --- | --- | --- | --- | --- | --- | --- | --- | --- | --- | --- | --- |
|  | | (cm) | (kg) | 3 | 10 | 25 | 50 | 75 | 90 | 97 |  |
| **V̇O_2peak_** **(L/min)** | | | |  |  |  |  |  |  |  |  |
| *Females* | |  |  |  |  |  |  |  |  |  |  |
| 55-59 | | 169 | 73.3 | 1.40 | 1.62 | 1.84 | 2.08 | 2.33 | 2.55 | 2.76 |  |
| 60-64 | | 168 | 69.9 | 1.27 | 1.49 | 1.71 | 1.95 | 2.20 | 2.42 | 2.63 |  |
| 65-69 | | 166 | 67.6 | 1.12 | 1.34 | 1.56 | 1.81 | 2.05 | 2.27 | 2.49 |  |
| 70-75 | | 165 | 66.6 | 1.00 | 1.22 | 1.44 | 1.69 | 1.93 | 2.15 | 2.37 |  |
|  | |  |  |  |  |  |  |  |  |  |  |
| *Males* | |  |  |  |  |  |  |  |  |  |  |
| 55-59 | | 183 | 87.4 | 2.43 | 2.64 | 2.86 | 3.11 | 3.35 | 3.57 | 3.79 |  |
| 60-64 | | 182 | 85.4 | 2.07 | 2.29 | 2.51 | 2.75 | 3.00 | 3.22 | 3.44 |  |
| 65-69 | | 180 | 83.9 | 1.84 | 2.06 | 2.28 | 2.52 | 2.77 | 2.99 | 3.21 |  |
| 70-75 | | 180 | 83.2 | 1.54 | 1.75 | 1.97 | 2.22 | 2.46 | 2.68 | 2.90 |  |
|  | |  |  |  |  |  |  |  |  |  |  |
| **WR_peak_ (W)** | | | |  |  |  |  |  |  |  |  |
| *Females* | |  |  |  |  |  |  |  |  |  |  |
| 55-59 | | 169 | 73.3 | 130 | 147 | 164 | 183 | 202 | 220 | 237 |  |
| 60-64 | | 168 | 69.9 | 118 | 135 | 152 | 171 | 190 | 208 | 225 |  |
| 65-69 | | 166 | 67.6 | 104 | 121 | 138 | 157 | 176 | 194 | 211 |  |
| 70-75 | | 165 | 66.6 | 92 | 109 | 126 | 145 | 164 | 182 | 199 |  |
|  | |  |  |  |  |  |  |  |  |  |  |
| *Males* | |  |  |  |  |  |  |  |  |  |  |
| 55-59 | | 183 | 87.4 | 225 | 242 | 260 | 279 | 298 | 305 | 332 |  |
| 60-64 | | 182 | 85.4 | 195 | 212 | 229 | 248 | 277 | 284 | 301 |  |
| 65-69 | | 180 | 83.9 | 171 | 188 | 205 | 225 | 244 | 261 | 278 |  |
| 70-75 | | 180 | 83.2 | 154 | 171 | 188 | 207 | 227 | 244 | 261 |  |
|  | |  |  |  |  |  |  |  |  |  |  |
| Abbreviations: CRF, cardiorespiratory fitness; V̇**O_2peak_**, oxygen uptake at peak exercise; WR_peak_, work rate at peak exercise.  ^a^ n = 632 for V̇O_2peak_ and WR_peak_, as 29 participants did not perform a maximal cardiorespiratory effort.  a  ^a^ for V̇O_2peak_, WR_peak_, and V̇O_2VAT_/V̇O_2peak_, n = 632 because 29 participants did not perform a maximal cardiorespiratory effort. | | | | | | | | | | | |

**Table 2 Additive models of submaximal CRF parameters (**V̇**O_2VAT_ and OUES) for females (n = 336) and males (n = 325) in the different age groups.**

| Age | Body height | | Body mass | Percentiles | | | | | | | |
| --- | --- | --- | --- | --- | --- | --- | --- | --- | --- | --- | --- |
|  | | (cm) | (kg) | 3 | 10 | 25 | 50 | 75 | 90 | 97 |  |
| **V̇O_2VAT_** **(L/min)** | | | |  |  |  |  |  |  |  |  |
| *Females* | |  |  |  |  |  |  |  |  |  |  |
| 55-59 | | 169 | 73.3 | 0.68 | 0.85 | 1.02 | 1.21 | 1.40 | 1.57 | 1.73 |  |
| 60-64 | | 168 | 69.9 | 0.66 | 0.83 | 1.00 | 1.19 | 1.38 | 1.55 | 1.71 |  |
| 65-69 | | 166 | 67.6 | 0.62 | 0.79 | 0.96 | 1.15 | 1.33 | 1.50 | 1.67 |  |
| 70-75 | | 165 | 66.6 | 0.57 | 0.74 | 0.91 | 1.09 | 1.28 | 1.45 | 1.62 |  |
|  | |  |  |  |  |  |  |  |  |  |  |
| *Males* | |  |  |  |  |  |  |  |  |  |  |
| 55-59 | | 183 | 87.4 | 1.05 | 1.22 | 1.39 | 1.57 | 1.76 | 2.93 | 2.10 |  |
| 60-64 | | 182 | 85.4 | 0.95 | 1.12 | 1.29 | 1.48 | 1.66 | 1.83 | 2.00 |  |
| 65-69 | | 180 | 83.9 | 0.90 | 1.07 | 1.24 | 1.43 | 1.61 | 1.78 | 1.95 |  |
| 70-75 | | 180 | 83.2 | 0.78 | 0.95 | 1.12 | 1.31 | 1.49 | 1.66 | 1.83 |  |
|  | |  |  |  |  |  |  |  |  |  |  |
| **OUES** | | | |  |  |  |  |  |  |  |  |
| *Females* | |  |  |  |  |  |  |  |  |  |  |
| 55-59 | | 169 | 73.3 | 1.39 | 1.68 | 1.98 | 2.32 | 2.65 | 2.95 | 3.26 |  |
| 60-64 | | 168 | 69.9 | 1.34 | 1.63 | 1.93 | 2.27 | 2.60 | 2.90 | 3.19 |  |
| 65-69 | | 166 | 67.6 | 1.21 | 1.51 | 1.80 | 2.14 | 2.47 | 2.77 | 3.06 |  |
| 70-75 | | 165 | 66.6 | 1.05 | 1.35 | 1.64 | 1.98 | 2.31 | 2.61 | 2.90 |  |
|  | |  |  |  |  |  |  |  |  |  |  |
| *Males* | |  |  |  |  |  |  |  |  |  |  |
| 55-59 | | 183 | 87.4 | 2.46 | 2.75 | 3.05 | 3.38 | 3.72 | 4.01 | 4.31 |  |
| 60-64 | | 182 | 85.4 | 2.25 | 2.54 | 2.84 | 3.17 | 3.51 | 3.80 | 4.10 |  |
| 65-69 | | 180 | 83.9 | 2.03 | 2.33 | 2.63 | 2.96 | 3.29 | 3.59 | 3.89 |  |
| 70-75 | | 180 | 83.2 | 1.86 | 2.15 | 2.45 | 2.79 | 3.12 | 3.42 | 3.71 |  |
|  | |  |  |  |  |  |  |  |  |  |  |
| Abbreviations: CRF, cardiorespiratory fitness; OUES, oxygen uptake efficiency slope; V̇**O_2VAT_**, oxygen uptake at the ventilatory anaerobic threshold. | | | | | | | | | | | |

**Table 3 Additive models of maximal CRF parameters (**V̇**O_2peak_ and WR_peak_) corrected for lean body mass for females (n = 323)^a^ and males (n = 309)^a^ in the different age groups.**

| Age | Body height | | Body mass | Percentiles | | | | | | | |
| --- | --- | --- | --- | --- | --- | --- | --- | --- | --- | --- | --- |
|  | | (cm) | (kg) | 3 | 10 | 25 | 50 | 75 | 90 | 97 |  |
| **V̇O_2peak_ (mL/kg lean body mass/min)** | | | | |  |  |  |  |  |  |  |
| *Females* | |  |  |  |  |  |  |  |  |  |  |
| 55-59 | | 169 | 73.3 | 36.0 | 39.8 | 43.7 | 48.1 | 52.4 | 56.3 | 60.2 |  |
| 60-64 | | 168 | 69.9 | 33.9 | 37.8 | 41.7 | 46.0 | 50.3 | 54.2 | 58.1 |  |
| 65-69 | | 166 | 67.6 | 31.8 | 35.7 | 39.6 | 43.9 | 48.3 | 52.2 | 56.0 |  |
| 70-75 | | 165 | 66.6 | 29.5 | 33.4 | 37.3 | 41.7 | 46.0 | 49.9 | 53.8 |  |
|  | |  |  |  |  |  |  |  |  |  |  |
| *Males* | |  |  |  |  |  |  |  |  |  |  |
| 55-59 | | 183 | 87.4 | 38.5 | 42.4 | 46.3 | 50.6 | 55.0 | 58.9 | 62.7 |  |
| 60-64 | | 182 | 85.4 | 35.2 | 39.0 | 42.9 | 47.3 | 51.6 | 55.5 | 59.8 |  |
| 65-69 | | 180 | 83.9 | 31.8 | 35.7 | 39.6 | 43.9 | 48.2 | 52.1 | 56.0 |  |
| 70-75 | | 180 | 83.2 | 28.4 | 32.2 | 36.1 | 40.5 | 44.8 | 48.7 | 52.6 |  |
|  | |  |  |  |  |  |  |  |  |  |  |
| **WR_peak_ (W/kg lean body mass)** | | | |  |  |  |  |  |  |  |  |
| *Females* | |  |  |  |  |  |  |  |  |  |  |
| 55-59 | | 169 | 73.3 | 3.32 | 3.62 | 3.91 | 4.34 | 4.58 | 4.87 | 5.17 |  |
| 60-64 | | 168 | 69.9 | 3.11 | 3.41 | 3.71 | 4.04 | 4.37 | 4.67 | 4.96 |  |
| 65-69 | | 166 | 67.6 | 3.90 | 3.20 | 3.49 | 3.83 | 4.16 | 4.46 | 4.75 |  |
| 70-75 | | 165 | 66.6 | 2.66 | 2.95 | 3.25 | 3.58 | 3.92 | 4.21 | 4.51 |  |
|  | |  |  |  |  |  |  |  |  |  |  |
| *Males* | |  |  |  |  |  |  |  |  |  |  |
| 55-59 | | 183 | 87.4 | 3.32 | 3.62 | 3.91 | 4.34 | 4.58 | 4.87 | 5.17 |  |
| 60-64 | | 182 | 85.4 | 3.11 | 3.41 | 3.71 | 4.04 | 4.37 | 4.67 | 4.96 |  |
| 65-69 | | 180 | 83.9 | 3.90 | 3.20 | 3.49 | 3.83 | 4.16 | 4.46 | 4.75 |  |
| 70-75 | | 180 | 83.2 | 2.66 | 2.95 | 3.25 | 3.58 | 3.92 | 4.21 | 4.51 |  |
|  | |  |  |  |  |  |  |  |  |  |  |
| Abbreviations: CRF, cardiorespiratory fitness; V̇**O_2peak_**, oxygen uptake at peak exercise; WR_peak_, work rate at peak exercise ^a^ n = 632 for V̇O_2peak_ and WR_peak_, as 29 participants did not perform a maximal cardiorespiratory effort. | | | | | | | | | | | |

**Table 4 Additive models of submaximal CRF parameters (**V̇**O_2VAT_ and OUES) corrected for lean body mass for females (n = 336) and males (n = 325) in the different age groups.**

| Age | Body height | | Body mass | Percentiles | | | | | | | |
| --- | --- | --- | --- | --- | --- | --- | --- | --- | --- | --- | --- |
|  | | (cm) | (kg) | 3 | 10 | 25 | 50 | 75 | 90 | 97 |  |
| **V̇O_2VAT_ (mL/kg lean body mass/min)** | | | | |  |  |  |  |  |  |  |
| *Females* | |  |  |  |  |  |  |  |  |  |  |
| 55-59 | | 169 | 73.3 | 18.1 | 21.2 | 24.4 | 27.8 | 31.3 | 34.4 | 37.5 |  |
| 60-64 | | 168 | 69.9 | 18.3 | 21.4 | 24.6 | 28.0 | 31.5 | 34.6 | 36.7 |  |
| 65-69 | | 166 | 67.6 | 18.2 | 21.3 | 24.4 | 27.9 | 31.4 | 34.5 | 37.6 |  |
| 70-75 | | 165 | 66.6 | 17.2 | 20.3 | 23.4 | 26.9 | 30.4 | 33.5 | 36.6 |  |
|  | |  |  |  |  |  |  |  |  |  |  |
| *Males* | |  |  |  |  |  |  |  |  |  |  |
| 55-59 | | 183 | 87.4 | 15.9 | 19.0 | 22.1 | 25.6 | 29.1 | 32.2 | 35.3 |  |
| 60-64 | | 182 | 85.4 | 15.2 | 18.3 | 21.4 | 24.9 | 28.4 | 31.5 | 34.8 |  |
| 65-69 | | 180 | 83.9 | 14.4 | 17.5 | 20.7 | 24.1 | 27.6 | 30.8 | 33.8 |  |
| 70-75 | | 180 | 83.2 | 12.6 | 15.7 | 18.9 | 22.3 | 25.3 | 29.0 | 32.0 |  |
|  | |  |  |  |  |  |  |  |  |  |  |
| **OUES/kg lean body mass** | | | |  |  |  |  |  |  |  |  |
| *Females* | |  |  |  |  |  |  |  |  |  |  |
| 55-59 | | 169 | 73.3 | 36.8 | 42.1 | 47.5 | 53.5 | 59.5 | 64.8 | 70.1 |  |
| 60-64 | | 168 | 69.9 | 36.7 | 42.0 | 47.4 | 53.3 | 59.3 | 64.7 | 70.0 |  |
| 65-69 | | 166 | 67.6 | 35.3 | 40.6 | 46.0 | 52.0 | 58.0 | 63.4 | 68.7 |  |
| 70-75 | | 165 | 66.6 | 35.6 | 37.4 | 42.8 | 48.7 | 54.7 | 60.1 | 65.4 |  |
|  | |  |  |  |  |  |  |  |  |  |  |
| *Males* | |  |  |  |  |  |  |  |  |  |  |
| 55-59 | | 183 | 87.4 | 38.2 | 43.5 | 48.9 | 54.9 | 60.8 | 66.2 | 71.5 |  |
| 60-64 | | 182 | 85.4 | 35.9 | 41.2 | 46.6 | 52.6 | 58.5 | 63.9 | 69.2 |  |
| 65-69 | | 180 | 83.9 | 33.5 | 38.8 | 44.2 | 50.1 | 56.1 | 61.5 | 66.8 |  |
| 70-75 | | 180 | 83.2 | 31.1 | 36.4 | 41.8 | 47.7 | 53.7 | 59.1 | 64.4 |  |
|  | |  |  |  |  |  |  |  |  |  |  |
| Abbreviations: CRF, cardiorespiratory fitness; OUES, oxygen uptake efficiency slope; V̇**O_2VAT_**, oxygen uptake at the ventilatory anaerobic threshold. | | | | | | | | | | | |

**
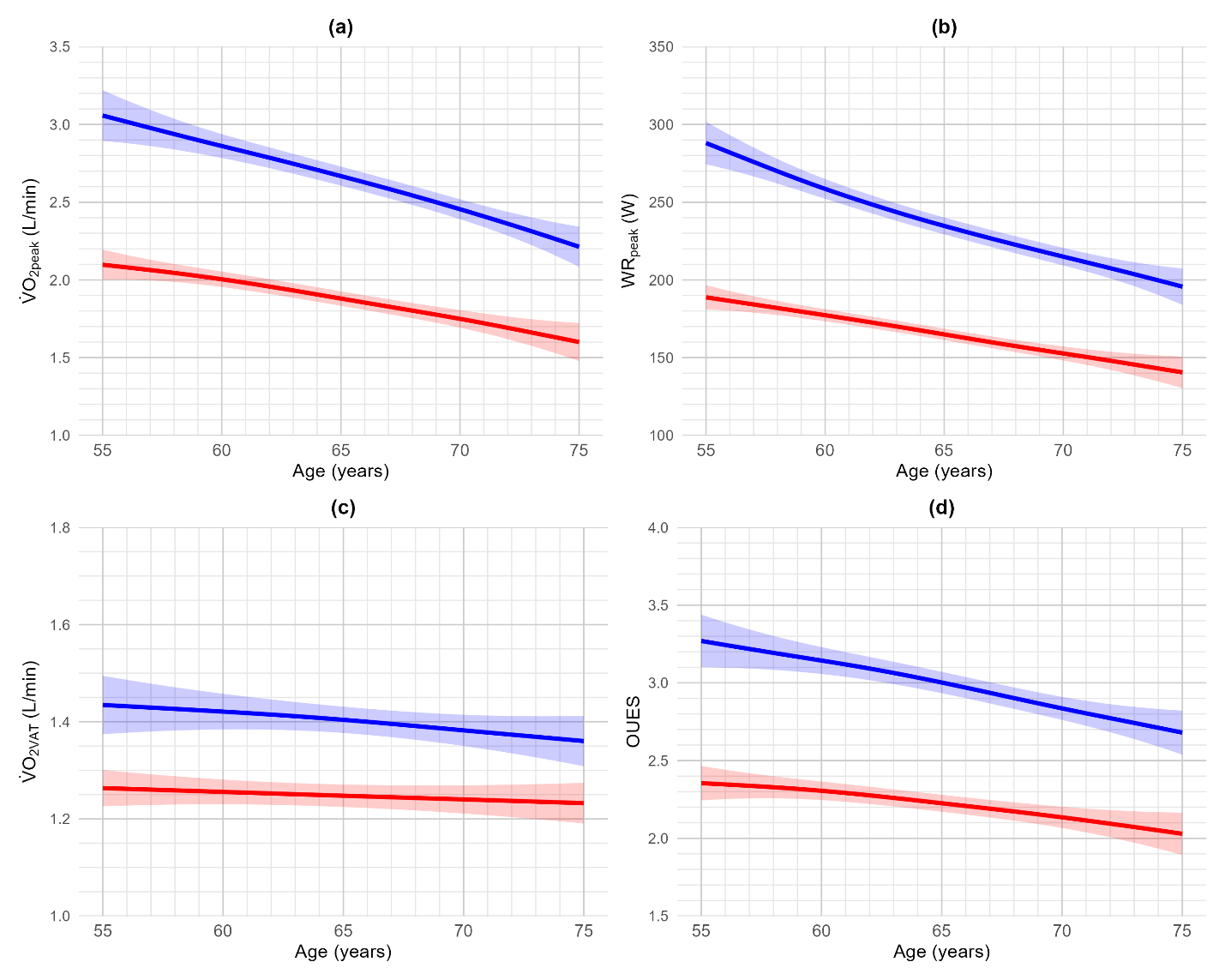
**

**Fig. 1 Cardiopulmonary exercise testing-derived, age-dependent transformation of a) oxygen uptake at peak exercise, b) work rate at peak exercise, c) oxygen uptake at the ventilatory anaerobic threshold and d) oxygen uptake efficiency slope in females (red) and males (blue) using generalized additive models. Shading represents 95% confidence intervals.** *OUES* oxygen uptake efficiency slope, *V̇O_2VAT_* oxygen uptake at the ventilatory anaerobic threshold, *V̇O_2peak_* oxygen uptake at peak exercise, *WR_peak_*, work rate at peak exercise


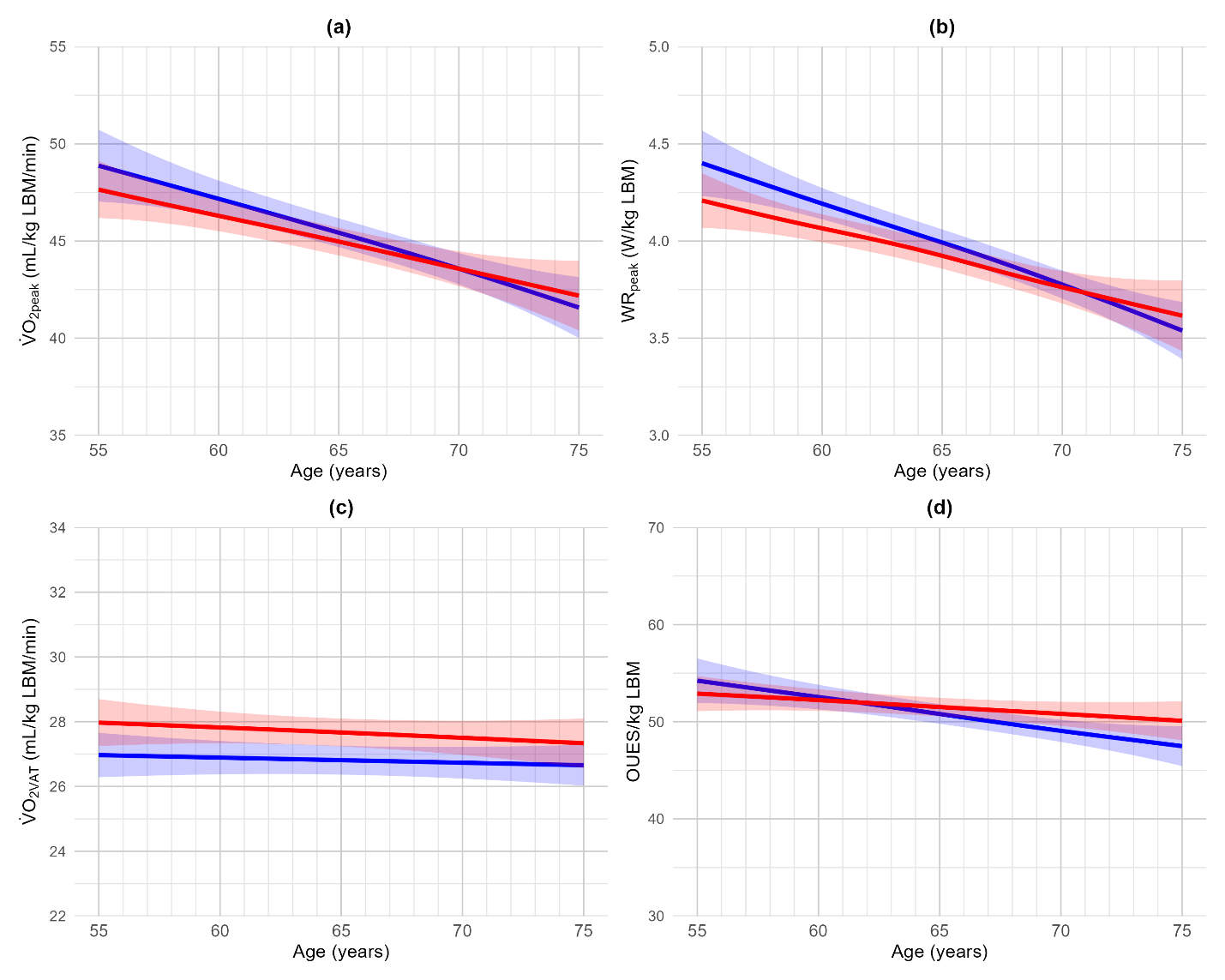


**Fig. 2 Cardiopulmonary exercise testing-derived, age-dependent transformation of a) oxygen uptake at peak exercise, b) work rate at peak exercise, c) oxygen uptake at the ventilatory anaerobic threshold and d) oxygen uptake efficiency slope corrected for lean body mass in females (red) and males (blue) using generalized additive models. Shading represents 95% confidence intervals.** *OUES* oxygen uptake efficiency slope, *V̇O_2VAT_* oxygen uptake at the ventilatory anaerobic threshold, *V̇O_2peak_* oxygen uptake at peak exercise, *WR_peak_*, work rate at peak exercise
